# The Omentum and Its Functions

**Published:** 1907-02

**Authors:** G. K. Dickinson

**Affiliations:** Jersey City. Annals of Surgery


*THE OMENTUM AND ITS FUNCTIONS.* G. K. Dickinson, Jersey City. Annals of Surgery, November, 1906.

The author reviews the embryology, anatomy and histology of the omentum, also sifting the detailed experimental literature dealing with this interesting organ. He draws the following conclusions:

The numerous bloodvessels and lax tissues of the omentum allow of storage of blood when the general arterial tension is high.

By venous anastomosis through adhesions, local congestion may be relieved.

Through its large surface freely exposed to surrounding parts in motion, it becomes a rapid absorber of fluids by the blood stream.

By the lymph stream it is a free carrier of white blood corpuscles, encapsulating soiled particles.

Through its cohesive tendency, apertures in the abdomen into which the omentum has been forced by intraabdominal pressure become more or less completely closed.

Through its readiness to lymph formation and local proliferation, it becomes attached to infected parts, which are walled off, subsequently to be absorbed by phagocytic action; the peritoneal cavity is thereby protected.

The majority of phagocytes extruded into the peritoneum for its protection come through the omentum, largely from the general circulation, but in part from the tissues therein existing; subsequently to be attached to the surface of this tissue, taken into the lymph-stream, and subjected to the cytolytic influence existing there.

Lack of development of the omentum, or loss through operation, renders one less resistant to peritoneal invasion.

Hemolymph glands of the splenic type existing in its base supplement the spleen if the latter be removed or its function interfered with.

